# KLHL14 and E-Cadherin Nuclear Co-Expression as Predicting Factor of Nonfunctioning PitNET Invasiveness: Preliminary Study

**DOI:** 10.3390/jcm13154409

**Published:** 2024-07-28

**Authors:** Jacopo Berardinelli, Valentina Russo, Angelo Canciello, Oriana Di Giacinto, Annunziata Mauro, Delia Nardinocchi, Ilaria Bove, Domenico Solari, Marialaura Del Basso De Caro, Luigi Maria Cavallo, Barbara Barboni

**Affiliations:** 1 Department of Neurosciences, Reproductive and Odontostomatological Sciences, Division of Neurosurgery, University of Naples “Federico II”, 80138 Naples, Italy; iacopobe96@gmail.com (J.B.); ilariabove90@gmail.com (I.B.); domenico.solari@unina.it (D.S.); lcavallo@unina.it (L.M.C.); 2 Department of Biosciences and Technology for Food, Agricultural and Environment, University of Teramo, 64100 Teramo, Italy; vrusso@unite.it (V.R.); odigiacinto@unite.it (O.D.G.); amauro@unite.it (A.M.); dnardinocchi@unite.it (D.N.); bbarboni@unite.it (B.B.); 3 Department of Advanced Biomedical Sciences, University of Naples “Federico II”, 80138 Naples, Italy; delbasso@unina.it

**Keywords:** pituitary adenoma, nonfunctioning pituitary adenomas, KLHL14, E-cadherin, epithelial–mesenchymal transition

## Abstract

**Background/Objectives.** Novel diagnostic and therapeutic approaches are needed to improve the clinical management of nonfunctioning pituitary neuroendocrine tumors (NF-PitNETs). Here, the expression of two proteins controlling the epithelial–mesenchymal transition (EMT)—an underlying NF-PitNET pathogenic mechanism—were analyzed as prognostic markers: E-cadherin (E-Cad) and KLHL14. **Methods.** The immunohistochemistry characterization of KLHL14 and E-Cad subcellular expression in surgical specimens of 12 NF-PitNET patients, with low and high invasiveness grades (respectively, Ki67^+^ < and ≥3%) was carried out. **Results.** The analysis of healthy vs. NF-PitNET tissues demonstrated an increased protein expression and nuclear translocation of KLHL14. Moreover, both E-Cad and KLHL14 shifted from a cytoplasmic (C) form in a low invasive NF-PitNET to a nuclear (N) localization in a high invasive NF-PitNET. A significant correlation was found between E-Cad/KLHL14 co-localization in the cytoplasm (*p* = 0.01) and nucleus (*p* = 0.01) and with NF-PitNET invasiveness grade. **Conclusions.** Nuclear buildup of both E-Cad and KLHL14 detected in high invasive NF-PitNET patients highlights a novel intracellular mechanism governing the tumor propensity to local invasion (Ki67^+^ ≥ 3%). The prolonged progression-free survival trend documented in patients with lower KLHL14 expression further supported such a hypothesis even if a larger cohort of NF-PitNET patients have to be analyzed to definitively recognize a key prognostic role for KLHL14.

## 1. Introduction

Pituitary neuroendocrine tumors (PitNETs) are among the most common primary intracranial tumors and their incidence is 10–20% of all brain tumors [1,2]. Pituitary tumors that do not produce biologically active hormones and do not exhibit specific hormonal symptoms are referred to as Nonfunctioning Pituitary Neuroendocrine Tumors (NF-PitNETs). NF-PitNETs make up approximately 30% of all PitNETs [3,4]. Although most PitNETs are benign, the incidence of invasive PitNETs has been reported to range from 35% to nearly 50% [1].

In particular, NF-PitNETs, due to their lack of hormonal symptoms, become clinically relevant when the tumor grows to surrounding tissues, invading the cavernous sinus and saddle bone, and oppressing the optic chiasma [5]. NF-PitNET aggressiveness can be predicted currently by exclusively combining clinical information with predictive markers of tumor proliferation (such as Ki-67) [6,7,8]. Therefore, predicting invasive behavior in PitNETs is challenging, leading to numerous studies focused on identifying potential biomarkers [9,10].

As an epithelial tissue, the pituitary gland is histologically classified through immunolabeling for epithelial markers, among other criteria (e.g., E-cadherin: E-Cad) [11]. The loss of the epithelial phenotype through a process known as epithelial–mesenchymal transition (EMT) is a hallmark of many tumors and is associated to an increased invasiveness, metastasis or poor prognosis [12,13]. Biologically, the loss of E-Cad at the cell surface is considered a key hallmark of EMT and is predictive of invasion and metastasis aggressiveness [14,15,16,17].

To date, many studies have confirmed that EMT plays an important role PitNET malignant transformation [18]. Moreover, the loss of E-Cad membrane localization and its nuclear translocation was associated to EMT in invasive NF-PitNETs [11,19].

To this regard, KLHL14 was recently identified as an emerging factor involved in the inhibition of EMT and in the pathogenesis of different tumors, even though nothing is known about PitNETs or NF-PitNETs [20,21,22,23,24,25].

Interestingly, the potential anti-EMT function of KLHL14 raises the possibility that its hypothetical target could be an EMT-associated protein, such as E-cadherin. To explore this hypothesis, the current study was designed to evaluate (1) KLHL14 expression in NF-PitNET specimens from patients with different grade of invasiveness, (2) E-Cad and KLHL14 subcellular localization, and (3) the correlation of E-Cad/KLHL14 expression and/or co-localization as predictive markers of NF-PitNET recurrence.

## 2. Materials and Methods

### 2.1. Survey Data Analysis of KLHL14 Expression in Tumors

The analysis of KLHL14 somatic gene mutation distribution for *n* = 228 cancer samples was acquired from the TCGA portal (https://portal.gdc.cancer.gov/) (up to 12 April 2024). Moreover, the TGCA portal was used to acquire information regarding the most mutated regions of the KLHL14 gene, and the frequency of copy number variation (CNV) cases (28) in relation to their position on the gene. Afterward, the distribution of KLHL14 mutations in 140 cases of brain tumor was analyzed along with the relative overall survival plot.

### 2.2. Patients and Samples

We performed an observational retrospective analysis on 12 consecutive patients who underwent endoscopic endonasal resection of an invasive NF-PitNET between January 2003 and September 2015 at the Division of Neurosurgery of the University of Naples “Federico II”. Clinical information, including basic information, preoperative and postoperative imaging examination, and pathological results, was gathered. Invasion was evaluated intraoperatively and classified as the breach and the extension by the lesion beyond either the medial wall of the cavernous sinus, the diaphragm sellae or the third ventricular floor. The grade of parasellar extension was assessed preoperatively based on the Knosp grading system and confirmed by intraoperative findings of a breach in the medial cavernous sinus wall. The main tumor extension and degree of supra and parasellar invasion for each patient is reported in Table 1. In addition, NF-PitNETs were also subclassified based on Ki67 positivity. To validate the antibodies for the immunohistochemical analyses, the expression of both E-cadherin (https://www.proteinatlas.org/ENSG00000039068-CDH1/tissue, accessed on 1 July 2024) and KLHL14 (https://www.proteinatlas.org/ENSG00000197705-KLHL14/tissue, accessed on 1 July 2024) was examined on The Human Protein Atlas. As a result, the thyroid gland was identified as a positive healthy control for both E-Cad and KLHL14. Consequently, two samples of healthy thyroid gland were purchased from BioChain Institute Inc., Newark, CA, USA. On the other hand, based on the TGCA results, human glioma and lung adenocarcinoma were employed as pathological control tissues to assess the ability of the antibody to detect nuclear KLHL14 translocation. For this purpose, mouse xenografts of either human glioma and lung adenocarcinoma were provided by the Pathological Anatomy Department at Teramo Hospital.

This observational study was conducted in accordance with the ethical standards set forth in the 1964 Declaration of Helsinki and its subsequent amendments or comparable ethical standards, and it is registered on ClinicalTrial.gov (Registration number NCT06247709). All the participants provided written informed consent for participation in this study.

### 2.3. Antibodies, Immunohistochemistry (IHC) and Confocal Microscopy Analyses

Formalin-fixed paraffin-embedded tissues serially sliced at 10 μm thicknesses were available from 12 patients diagnosed with an NF-PitNET based on the classification using standard hematoxylin–eosin and reticulin staining, periodic acid–Schiff (PAS) and detailed immunohistochemical analysis, for anterior pituitary hormones carried out at the laboratory of Pathological Anatomy of the department of advanced biomedical sciences of the University of Naples “Federico II”. In addition, this laboratory analyzed the expression of Ki67 in all cases by using the monoclonal antibody MIB-1 Immunotech (dilution 1:100, overnight incubation).

Before E-Cad and KLHL14 IHC staining, dewaxing and alcohol hydration was performed on a tissue section of healthy and pathological tissues.

Firstly, single IHCs were performed to verify the specificity of the antibodies. In brief, heat-induced epitope retrieval was conducted for 10 min at 95 °C. Non-specific binding was blocked, incubating the sections at room temperature (RT) in 1% bovine serum albumin (BSA) in PBS for 1 h. Tissue sections were incubated overnight (O/N) at RT with 1:50 Anti-KLH14 (PA5-107155; Invitrogen, Waltham, MA, USA) and 1:200 Anti-E-Cad (CDH1) (ABIN1440031; Antibodies-online), recognizing its cytoplasmic domain [26]. Subsequently, sections were exposed respectively to 1:200 Anti-Rabbit IgG antibody—CY3 (Merck Millipore, Burlington, MA, USA; AP132C) and 1:200 Anti-Rabbit IgG antibody—Alexa Fluor^®^ 488 (Abcam, Cambridge, UK; ab150077) for 40 min at RT. To verify E-Cad and KLHL14 co-localization, a double-labeling was carried out according to Russo et al. (2015) [27]. In brief, non-specific binding was blocked, incubating the sections as specified before. Sections were then incubated with the rabbit anti-E-Cad at RT-O/N. After washing, this immunocomplex layer was detected by an anti-rabbit Ab Alexafuor488 conjugated. Furthermore, after washing in PBS/1%BSA, the same sections were firstly fixed for 10 min with 4% paraformaldehyde/PBS, and then, after washing with PBS, incubated with a rabbit anti-KLHL14 at RT-O/N. After washing in PBS, tissue sections were then processed with a Cy3-labeled secondary goat anti-rabbit Ab. At the end of each immunoreaction, DNA was counterstained with DAPI (Sigma, St. Louis, MO, USA) diluted 1:100 in PBS for 10 min. In all experiments, non-immune serum was used in place of the primary antisera as a negative control. All controls performed were negative. At least 15 different sections for each sample were observed under an AIR laser confocal scanning microscope (Nikon, Düsseldorf, Germany), using a Plan Apo 40x oil objective. The used channels were: Channel 1: DAPI; λexc = 405 nm; Channel 2: FITC; λexc = 488 nm; and Channel 3: TRITC; λexc = 561 nm. Optimum exposure times were determined and held constant thereafter. The immune-stained sections were studied on reconstructed images made by projecting 1 lm Z-stacks of two or three consecutive confocal images. The laser confocal scanning microscope was equipped with NIS-Element software 4.40 (Nikon, Düsseldorf, Germany) used as a program for fluorescence analysis on unaltered digitalized images. Two different observers performed the qualitative assessment of immunostaining distribution and immune-fluorescence co-localizations. The three observers were blinded as to other information and considered the immune-fluorescence positivity based on its intensity in high and low and recorded the fluorescence localization giving priority to the nuclear one when both cell compartments (cytoplasm and nucleus) were stained.

The IHC staining for E-Cad and KLHL14 was then scored using the immunoreactive score (IRS), as previously described [28]. IRS is the product of the proportion of immunoreactive cells (B) and the staining intensity (A) (“high”, “low” or “no staining”). Consequently, the IRS score (which can range from 0 to 10) was classified as “low” with values < 5 or “high” with values ≥ 5.

### 2.4. Statistical Analysis

Categorical variables are reported as absolute numbers and percentages, whereas continuous variables are reported as median value ± standard deviation. The χ^2^ test was used to analyze the relationship between the IRS of E cadherin and KLHL14, while a two-tailed unpaired Mann–Whitney U test was applied to compare the difference in frequency of expression of E-cadherin and KLHL14 within groups with high and low Ki67. Log-rank analysis was used to determine the significance of the Kaplan–Meier survival curve. A *p*-value < 0.05 was considered significantly different. Raw data were entered into Microsoft Excel (Version 16.63.1 for Mac). Statistical analysis was performed with R (version 4.3.0, The R Foundation for Statistical Computing) and RStudio (Version 2023.06.0+).

## 3. Results

### 3.1. KLHL14 Survey in Tumors

**Figure 1:**
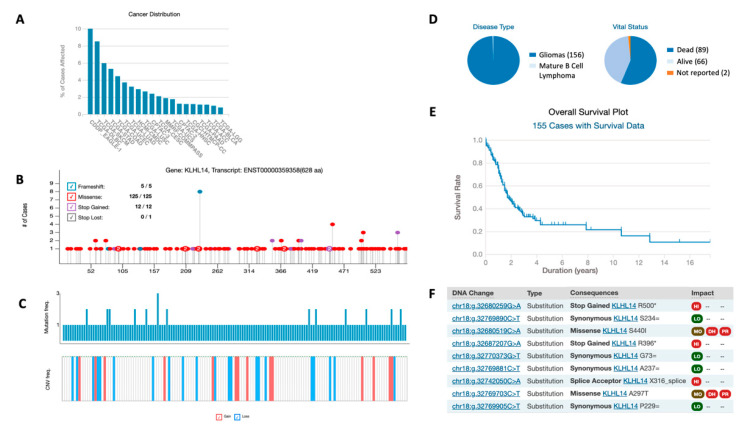
KLHL14 mutations and distribution in cancers. (**A**) This graph shows the percentage of tumor cases affected by KLHL14 mutations and their distribution in the different types of cancer. In particular, TGCA database reported 226 different types of mutations in 231 tumor cases. (**B**) Graphical representation of KLHL14 genes and the localizations of all the mutations reported in TGCA database. (**C**) The upper part reports the graphical representation of KLHL14 mutation frequency and the lower part, the frequency of KLHL14 copy number variation (CNV) cases (28) in relation to their position on the gene. In particular, the lower part reports the CNV that led to gain events, while in blue are those that led to loss events. (**D**) Distribution of 155 cases of brain tumor associated with KLHL14 according to TCGA and (**E**) the relative overall survival plot. (**F**) The six cases of KLHL14 mutations found in brain tumor and the relative description. All the information about KLHL14 gene was collected from TCGA database.

Preliminarily, the analysis of KLHL14 mutations’ involvement in different types of cancer was performed by using the TCGA portal. The results indicated that 226 type of mutations were identified in 231 different tumor cases (Figure 1A). Regarding the distribution, KLHL14 mutations were mainly (10% of the cases) found in adenomas and adenocarcinomas, followed by lymphoma (8.51% of the cases) and melanoma (5.96% of the cases) (Figure 1A). Only in 0.78% of the cases were KLHL14 mutations found in brain tumors; specifically, in lower grade glioma (Figure 1A). At present, no information can be found for IPitNETs and NF-PitNETs. Intriguingly, the analysis of mutation distribution along KLHL14 showed that all the regions of this gene are equally hit by mutation events (Figure 1B). Similarly, KLHL14 copy number variation (CNV) (*n* = 28 cases) analysis showed that all the functional domains are involved in either gain and loss events (Figure 1C). Finally, the distribution of KLHL14 mutations was evaluated in 155 cases of brain tumor along with a relative overall survival plot (Figure 1D,E). However, only six different types of KLHL14 mutations were found (Figure 1F).

### 3.2. E-Cadherin and KLHL14 Antibodies Validation

The E-Cad and KLHL14 antibodies were tested on sections of both healthy and cancer tissues for single and double IHC reaction. The specificity of the KLHL14 and E-Cad antibody was, firstly, confirmed on a healthy thyroid where both the proteins displayed an exclusive cytoplasm localization (see Figure S1 Supplement). Subsequently, based on the TGCA results, NOD/SCID mouse xenografts of both human glioma and lung adenocarcinoma were used to detect the nuclear protein domain specificity of KLHL14 and E-Cad antibodies (see Figure S1 Supplement). As a result, cells showing E-Cad nuclear translocation were recorded in both cancer tissues (see Figure S1 Supplement). Of note, KLHL14 also acquired the nuclear positivity in both xenograft explants (see Figure S1 Supplement). Surprisingly, the contemporary immunostaining for KLHL14 and E-Cad in the xenografted tumor explants revealed the co-localization of these two proteins in both nuclear and cytoplasmic compartments (see Figure S2 Supplement).

### 3.3. Sample Characteristics

The mean age of NF-PitNET patients at diagnosis was 57.5 ± 9.5 years. The cohort included seven males (aged 44–70 years) and five females (aged 38–64 years). On preoperative imaging, four patients were classified a Knosp grade = 2, five as a Knosp grade 3 and two patients as Knosp grade 4. Cavernous sinus invasion was confirmed intraoperatively for all patients. All patients underwent maximal safe resection, with the main residual tumor intentionally left in the cavernous sinus. Two patients received radiotherapy as adjuvant treatment post-surgery, and none received neoadjuvant treatment with dopamine agonists before surgery. The mean recurrence time was 4.26 years. All tumors were clinically classified as nonfunctioning at the time of surgery and confirmed through histopathological analysis. The main clinical and histopathological data are summarized in Table 1. In accordance with WHO guidelines [8], NF-PitNET patients were categorized for analysis based on Ki67^+^ expression levels (Ki67 < 3% and >3%) [6].

### 3.4. Immunofluorescence Subcellular Distribution of E-Cadherin and KLHL14 in Healthy and NF-PitNET

**Figure 2:**
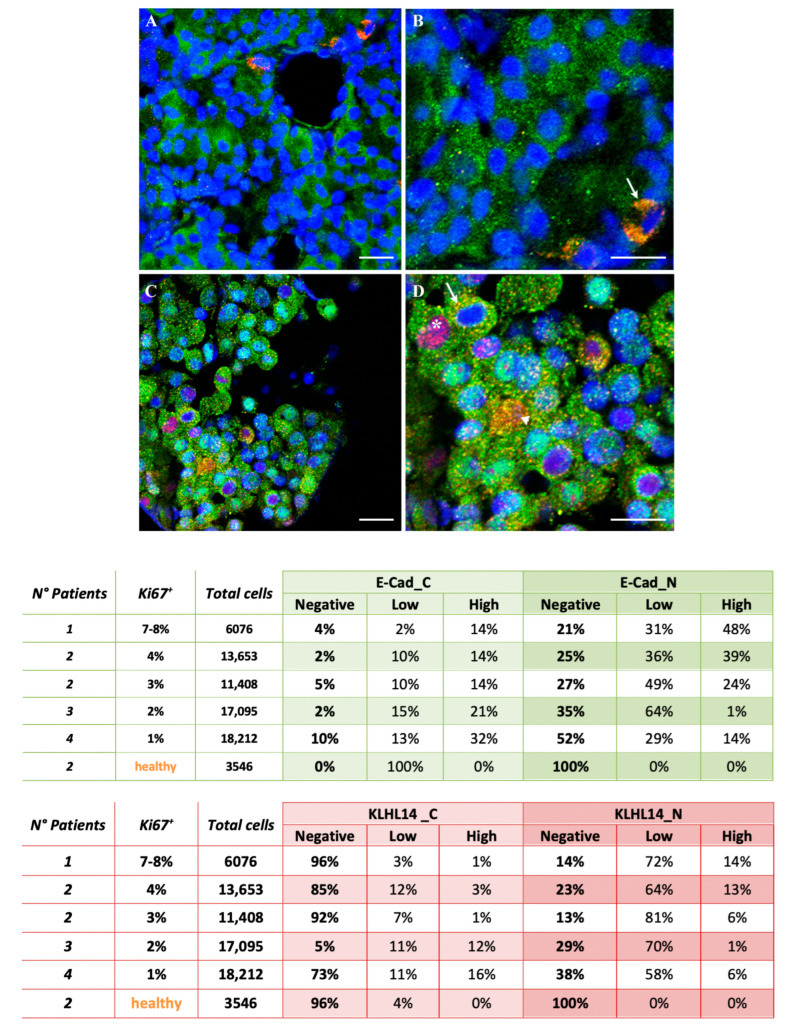
KLHL14 and E-Cadherin distribution and co-localization on healthy adenohypophysis or NF-PitNET tissue sections. Qualitative and quantitative assessment of cytoplasmic and nuclear location of E-Cad (Green table) and KLHL14 (Red table) in NF-PitNET and healthy tissue carried out with IF. Abbreviation: E-Cad_C and E-Cad_N: cytoplasm and nucleus E-cadherin positivity, respectively; KLHL14_C and KLHL14_N: KLHL14 cytoplasm and nucleus positivity, respectively; Ki67^+^: percentage of Ki67 positivity. Top images: Panels (**A**,**B**) are representative images of healthy adenohypophysis at different magnifications immune labelled for E-Cad and KHL14 ((**B**): higher magnification). The fluorescent signal allowed us to distinguish KLHL14 positivity in red, E-Cad in green and their co-localization in orange/yellowish (overlapping of the red and green fluorescent signals), while the nuclei are blue for DAPI counterstaining (blue fluorescence). These images clearly show the prevalence of cells displaying cytoplasmic E-Cad positivity and few co-localizations with KLHL14 (arrow). Top images: Panels (**C**,**D**) are similarly representative merged fluorescence images of NF-PitNET-derived tissue ((**D**): higher magnification). The KLHL14 and E-Cad positivity frequently co-localized (yellow/orange fluorescence) in both cytoplasm (arrow) and nuclei (arrowhead). Several cells displayed E-cadherin (green fluorescence) localized within the cytoplasm and KLHL14 (red fluorescence) in the nucleus (asterisk *). Scale bars: 25 μm. In the table, three independent observers quantified the subcellular positivity for E-Cad (E-Cad_C and E-Cad_N) and KLHL14 (KLHL14_C and KLHL14_N). Cells were classified based on the protein localization and on the immunofluorescence signal intensity (negative, low or high). When both cell compartments (cytoplasm and nucleus) were stained, priority was given to nuclear localization.

Healthy pituitary sections displayed cells with cytoplasmic E-Cad staining, while most were either KLHL14 negative or showed cytoplasmic positivity (Figure 2). In contrast, E-Cad and KLHL14 staining patterns significantly changed in NF-PitNET samples, revealing diverse protein locations as described in Figure 2. These included single E-Cad (green fluorescence) and KLHL14 (red fluorescence) positivity in the nucleus or cytoplasm, cytoplasmic co-localization of E-Cad and KLHL14 (yellow/orange fluorescence), and nuclear co-localization of E-Cad and KLHL14 (yellow/orange fluorescence), with the latter being the predominant fluorescence pattern in NF-PitNET tissues.

More specifically, the NF-PitNET biopsy samples showed a loss of cytoplasmic positivity for E-cadherin (E-Cad_C) in a Ki67^+^-dependent manner, favoring the co-localization patterns summarized in Figure 2 (Green Table). E-Cad_C positivity, which characterized all healthy adenohypophysis cells, decreased from approximately 40% in low Ki67^+^ NF-PitNET patients (out of 35,307 recorded cells) to 20% in high Ki67^+^ patients (out of 31,137 cells analyzed) (*p* = 0.01). Conversely, nuclear E-Cad (E-Cad_N) was more prevalent in high Ki67^+^ NF-PitNET patients with an incidence of 76% compared to 56% of low Ki67^+^ NF-PitNET patients (*p* = 0.01).

A similar relationship was observed for KLHL14, which showed increased expression and prominent nuclear translocation in high Ki67^+^ NF-PitNET patients (Figure 2). In particular, cytoplasmic KLHL14 (KLHL14_C) was lower in high Ki67^+^ patients (9%) than in low Ki67^+^ patients (25%) (*p* = 0.02) (Figure 2, Red Table). Conversely, the main KLHL14 immunopositivity of NF-PitNET cells was recorded in the nucleus (KLHL14_N), with an incidence ranging between 67% and 83% in low and high Ki67^+^ patients, respectively (*p* = 0.02).

When analyzing the different expressions of E-cadherin and KLHL14 with respect to preoperative Knosp grading, no statistically significant differences were found. However, a trend was noted with higher cytoplasmic localization among patients with Knosp ≤ 2 (E-Cad_C 39% vs. 29%, *p* = 0.29; KLHL14_C 22% vs. 15%, *p* = 0.34), whereas nuclear expression was more frequent in patients with Knosp grade ≥ 3 (E-Cad_N 66% vs. 53%, *p* = 0.20; KLHL14_N 75% vs. 70%, *p* = 0.51).

### 3.5. E-Cadherin and KLHL14 Immunoreactive Score (IRS) in NF-PitNET Patients

**Figure 3:**
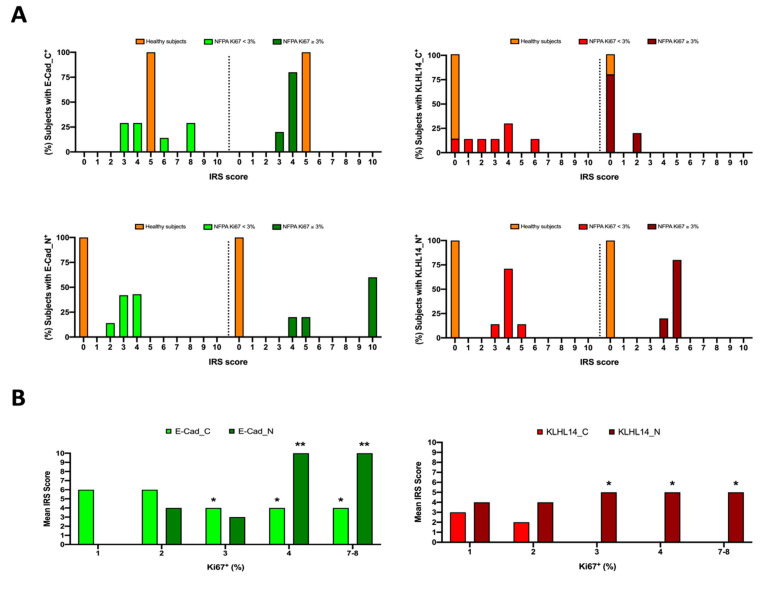
NF-PitNET patient distribution according to IRS score and Ki67 positivity. Panel (**A**) NF-PitNET patient distribution among cytoplasm (C) and nuclear (N) E-cadherin (E-Cad) and KLHL14 immunoreactive score categories (IRS). The IRS score categories (from 0 to 10) are described along the *x*-axis. The green and red bars indicate the % of NF-PitNET patients with low and high Ki67 expression belonging to each IRS categories of E-Cad or KLHL14 positivity (*y*-axis). The orange histogram identifies the IRS score category representative of healthy patients. Low and high IRS score are considered for categories < 5 and ≥5, respectively. Panel (**B**) Mean nuclear and cytoplasmic E-Cad and KLHL14 immunoreactive scores (IRS) in NF-PitNET patients with low and high Ki67 positivity. The mean values of IRS scores for E-Cad or KLHL14 are described along the *y*-axis inside NF-PitNET patients classified in x-axis on the basis of Ki67^+^ expression. S.D. never exceeds 5%. In each graph, statistical significance symbols (* *p* < 0.05; ** *p* < 0.01) indicate differences between the same expression pattern category (cytoplasm vs. cytoplasm or nucleus vs. nucleus localization).

The expression of E-Cad and KLHL14 was analyzed using the immunoreactive semiquantitative score (IRS) (see Section 2.3). Specifically, the IHC data were used to assign each patient a C or N_IRS score category at for both E-Cad^+^ and KLHL14^+^ (Figure 3). Notably, the distribution of IRS score categories differed between healthy and NF-PitNET patients and varied with Ki67^+^ expression in NF-PitNET patients. Most NF-PitNET patients were distributed on a higher IRS score category of E-Cad_N and KLHL14_N (Figure 3B compared to healthy individuals (IRS category = 0 for both proteins). In particular, most high Ki 67^+^ NF-PitNET patients had an IRS score category > 4.

Conversely, the IRS score categories for E-Cad_C and KLHL14_C showed a different distribution (Figure 3A). All NF-PitNET patients had a different IRS score category for E-Cad_C compared to healthy individuals (IRS score category = 5). Specifically, half of the low Ki67^+^ patients and all high Ki67^+^ patients were in lower IRS score categories (IRS score categories < 5: Figure 3A).

Differently, for KLHL14_C, the IRS score category distribution changed exclusively in low Ki67+ NF-PitNET patients, where most patients fell into higher IRS score categories (IRS categories > 0: Figure 3A), whereas nearly all high Ki67+ patients (75%) shared the same IRS score category as healthy subjects (Figure 3A)

The subcellular translocation of E-Cad or KLHL14 protein was analyzed by comparing the mean incidence of C_ and N_ IRS score categories in NF-PitNET patients (Figure 3B) according to their Ki67^+^ expression. This semiquantitative approach proved that E-Cad_C was always present in NF-PitNET tissues with the highest IRS score (IRS ≥ 5: *p* < 0.05) in low Ki67^+^ patients. On the contrary, E-Cad_N began to appear exclusively in NF-PitNET patients with Ki67^+^ ≥ 2%, reaching the highest IRS scores in patients with 4% and 7–8% Ki 67^+^ expression (for both *p* < 0.01 vs. 1% and 2% Ki67^+^: Figure 3B). In contrast, KHL14_C expression was observed exclusively in low Ki67^+^ patients (mean IRS score < 3: Figure 3B), disappearing in high Ki67^+^ patients. Finally, the mean incidence of the KHL14_N IRS score indicated its nuclear presence in all NF-PitNET patients, with a significantly higher mean IRS score in high Ki67^+^ patients (mean IRS score in low vs. high Ki 67^+^: *p* < 0.05).

### 3.6. E-Cadherin and KLHL14 Co-Localization in NF-PitNET Patients

**Figure 4:**
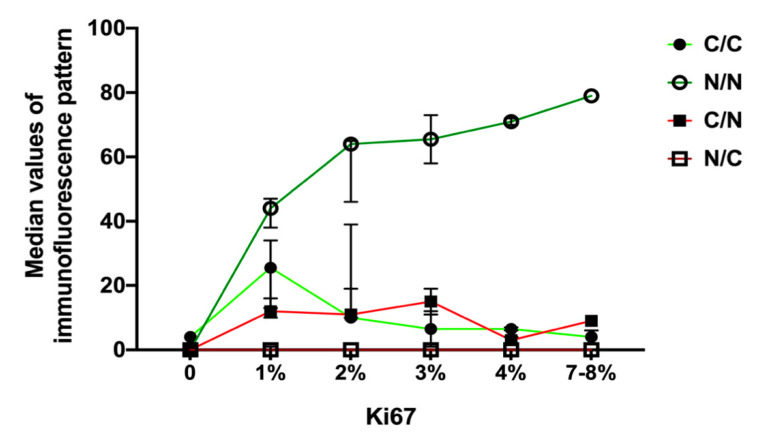
Incidence of E-Cadherin and KLHL14 immunofluorescence patterns in NF-PitNET patients with different Ki67 expression. The median values of immunofluorescence pattern are described along the *y*-axis inside NF-PitNET patients classified in x-axis on the basis of Ki67^+^ expression. The healthy patients belong to “Ki67^+^ = 0” category. Legend abbreviations: C/C: E-Cad_Cytoplasm/KLHL14_Cytoplasm; N/N: E-Cad_Nuclear/KLHL14_Nuclear; C/N: E-Cad_Cytoplasm/KLHL14_Nuclear; N/C: E-Cad_Nuclear/KLHL14_ Cytoplasm.

The incidence of E-Cad/KLHL14 co-localization patterns was analyzed according to Ki 67^+^ and is summarized in Figure 4. Specifically, the following co-localization patterns were considered in this analysis: E-Cad_C/KLHL14_C (C/C); E-Cad_N/KLHL14_N (N/N); E-Cad_C/KLHL14_N (C/N); and E-Cad_N/KLHL14_C (N/C). The analysis revealed a low occurrence of exclusive nuclear or cytoplasmic E-Cad and KHL14 immunofluorescence (less than 10% of total cells), regardless of Ki67^+^ levels (Figure 4). In contrast, the C/C or N/N co-localization patterns, which are predominant in NF-PitNET tissues, showed opposite trends. The overall frequency of C/C co-localization was significantly lower in patients with high Ki67^+^ compared to those with low Ki67^+^ (5.6% vs. 22.4%, *p* = 0.01). Conversely, N/N co-localization was significantly more frequent in high Ki67^+^ patients (70.4% vs. 49.6%, *p* = 0.01) (Figure 4).

### 3.7. KLHL14 Nuclear Expression and Correlation with Progression-Free Survival (PSF)

Based on different levels of nuclear KLHL14 expression, the NF-PitNET samples were divided into two subgroups: a high expression (*n* = 6) and low-expression group (*n* = 6). We then analyzed progression-free survival (PFS) between these subgroups using a Kaplan–Meier survival analysis. The results showed that patients with lower KLHL14 expression had prolonged PFS, but the survival advantage did not reach statistical significance in the log-rank test, likely due to the small sample size. The median PFS was 55.86 months in the low-expression group, compared to 26.0 months in the high-expression group (*p* = 0.14; Supplementary Figure S3).

## 4. Discussion

NF-PitNETs are the most common pituitary tumors though still lack effective therapeutic strategies [29,30]. The involvement of the diaphragm sellae and parasellar cavernous space significantly increases surgical complexity and the risk of intra- and postoperative complications. Therefore, identifying specific molecular targets of invasion in NF-PitNETs is crucial for developing more precise biological prognostic models and targeted medical treatments. This study focused on investigating KLHL14 expression, a promising new EMT marker, in NF-PitNET patients with varying grades of invasiveness.

Preliminary interrogation of the TCGA database indicated that although KLHL14 mutations are reported in various tumors, there is no information on its role in invasive PitNETs or NF-PitNETs. However, KLHL14 has a high heterogenic mutational rate with no specific hotspots identified, as all protein domains are equally affected. The absence of specific recurrent mutations makes it challenging to use this information for a screening purpose.

Therefore, we focused on the specific function of KLHL14 in tumorigenesis and EMT, a crucial event for NF-PitNET pathogenesis due to its association with increased malignancy and invasiveness [19,31,32]. EMT is not a binary process since an undefined number of intermediate cells with hybrid phenotypes can generate throughout the trans-differentiation [33]. As a consequence, EMT causes dramatic transcriptional and post-translational modifications which have been demonstrated to specifically interfere with NF-PitNET drug mechanisms of action [31,34,35].

KLHL14 was originally described as an interactor of Torsin A1 [36], and later studies found its involvement in axonal or B cell development [37,38]. Subsequently, KLHL14 has also been reported to act either as a tumor promoter, in ovarian and endometrial cancers, or a tumor suppressor, in a subtype of diffuse large B cell lymphoma [22,23,39]. However, the specific biological role of KLHL14 in tumorigenesis remains unclear. Recently, KLHL14 has been identified as a key protein in regulating the EMT process. Initially, KLHL14 was reported among the most upregulated genes involved in EMT inhibition, using amniotic epithelial cells (AECs) as a physiological model of EMT [24]. Then, it was demonstrated that KLHL14 possesses an anti-oncogenic action due to its ability to inhibit cell proliferation, migration, invasion, colony formation and EMT in malignant mesothelioma (MM) [25]. Similarly, KLHL14 was found to also act as a tumor suppressor in thyroid cancer, where it impairs cell growth, alters the expression of key thyroid differentiation markers and increases apoptosis in thyroid neoplastic cells [40].

This preliminary report documented for the first time the expression of KLHL14 in NF-PitNET samples, showing limited cytoplasmic expression in healthy subjects and widespread nuclear expression in pathological tissues. Furthermore, the nuclear localization of KLHL14 was significantly associated with higher tumor invasiveness grades. Previous studies have found KLHL14 originally located in the endoplasmic reticulum and, to a lesser extent, in the nuclear envelope [36]. However, clear nuclear expression of KLHL14 was reported in MM and thyroid cancer as well as in other cell lines [25,40]. In particular, it was demonstrated that KLHL14 subcellular localization changed depending on MM subtype: cytoplasmic expression was prevalent in epithelial subtypes, whereas nuclear expression was predominant in mixed and sarcomatoid phenotypes [25]. These results seem to suggest that nuclear localization could be associated with either changes in cell phenotype or malignant transformation, as frequently observed during EMT.

Importantly, KLHL14 is a Kelch-like (KLHL) family member, an evolutionarily conserved group of protein involved in the ubiquitination process [41]. To date, the specific ligand/s of KLHL14 is yet unknown, and for this reason, it could either be cytoplasmic or nuclear protein/s. Moreover, KLHL14’s anti-oncogenic and anti-EMT role might suggest that nuclear translocation could represent a deregulation of its normal functions. However, further studies are needed to elucidate KLHL14’s specific functions.

The involvement of KLHL14 in EMT led us to explore the hypothesis that its expression could somehow be related to other important proteins of this process. For this reason, we decided to investigate in NF-PitNET samples the expression of E-cadherin (E-Cad), the epithelial marker whose nuclear translocation is involved in EMT initiation [42]. Similar to KLHL14, E-Cad also showed only cytoplasmic positivity in healthy samples, with malignant transformation linked to increased nuclear expression and decreased cytoplasmic positivity. Intriguingly, nuclear localization was even higher in NF-PitNET patients with the highest invasiveness grade, confirming the results from Elston et al. [11]. Notably, KLHL14 showed an early nuclear translocation compared to E-Cad. In the literature, the aberrant nuclear expression of E-Cad has been reported in different types of cancers and there are numerous pieces of evidence of its correlation with malignant transformation and/or the metastatic process [43,44,45]. In agreement with our findings, nuclear accumulation of E-Cad has also recently been documented in NF-PitNETs and invasive PitNETs [11,46]. However, to the best of our knowledge, the present study represents the first report of an aberrant nuclear expression of E-Cad with KLHL14 in NF-PitNETs.

In conclusion, we have documented here a specific co-localization of KLHL14 and E-Cad signals in NF-PitNET samples. Notably, this co-localization was sensibly increased in the most invasive forms of this tumor, thus confirming a correlation of the gravity of NF-PitNETs with the nuclear translocation of KLHL14/E-Cad. The co-localization of these two proteins seems to occur in both the cytoplasmic and nuclear compartment with a precise sequence but to a different extent. Indeed, we have found a low cytoplasmic KLHL14/E-Cad co-localization in healthy or low invasive NF-PitNET samples. Conversely, nuclear KLHL14/E-Cad co-localization significantly increased and reached its peak in high invasive NF-PitNET samples, suggesting that the clear nature of this interaction has to be further elucidated. However, further molecular experiments are needed in order to confirm a direct or indirect KLHL14/E-cadherin interaction and to specifically demonstrate its biological role in EMT cellular models.

Given their silent state, NF-PitNETs are generally identified due to a mass effect rather than a specific hormonal symptom [47]. Therefore, if confirmed, nuclear translocation of KLHL14 with E-cadherin could represent a novel biomarker of tumor EMT as well as a potential therapeutic target to block adenoma invasion of suprasellar and parasellar structures, which represents one of the most important characteristics that increases the surgical degree of difficulty and often results in an incomplete extent of resection, therefore leading to an increased frequency of recurrent disease [48,49].

## Figures and Tables

**Table 1:** Main demographical, radiological and pathological information of NF-PitNET patients.

Case	Gender	Age (y)	Time of Recurrence (y)	Prevalent Extension	Knosp Grade	Invasion	Ki67	NFPA Histotype
1	M	44	4	Suprasellar	2	Cavernous Sinus	4%	High Ki67^+^ (≥3%)
2	M	56	4	Suprasellar	3	III Ventricle / Cavernous Sinus	4%	
3	F	59	1	Parasellar	4	Cavernous Sinus	7–8%	
4	F	38	5	Suprasellar	2	Paninvasive	3%	
5	M	70	1	Parasellar	3	Cavernous Sinus	3%	
6	F	54	3	Suprasellar	2	III Ventricle / Cavernous Sinus	2%	Low Ki67^+^ (<3%)
7	M	61	12	Suprasellar	3	Paninvasive	2%	
8	F	51	1	Parasellar	3	Cavernous Sinus	2%	
9	M	63	1	Suprasellar	2	Cavernous Sinus	2%	
10	M	68	5	Suprasellar	2	Paninvasive	1%	
11	M	62	2	Suprasellar	4	Paninvasive	1%	
12	F	64	4	Suprasellar	3	III Ventricle/Cavernous Sinus	1%	
13	F	n.d	--	--		--	<0.1%	Healthy
14	M	n.d	--	--		--	<0.1%	

## Data Availability

The datasets generated and/or analyzed during the current study are available from the corresponding author on reasonable request.

## Supplementary Materials

The following supporting information can be downloaded at: https://www.mdpi.com/article/10.3390/jcm13154409/s1.
