# Supplementary figures and images for: KLHL14 and E-Cadherin Nuclear Co-Expression as Predicting Factor of Nonfunctioning PitNET Invasiveness: Preliminary Study
Source: J Clin Med. 2024 Jul 28;13(15):4409. doi: 10.3390/jcm13154409 (PMC11312959; doi:10.3390/jcm13154409)

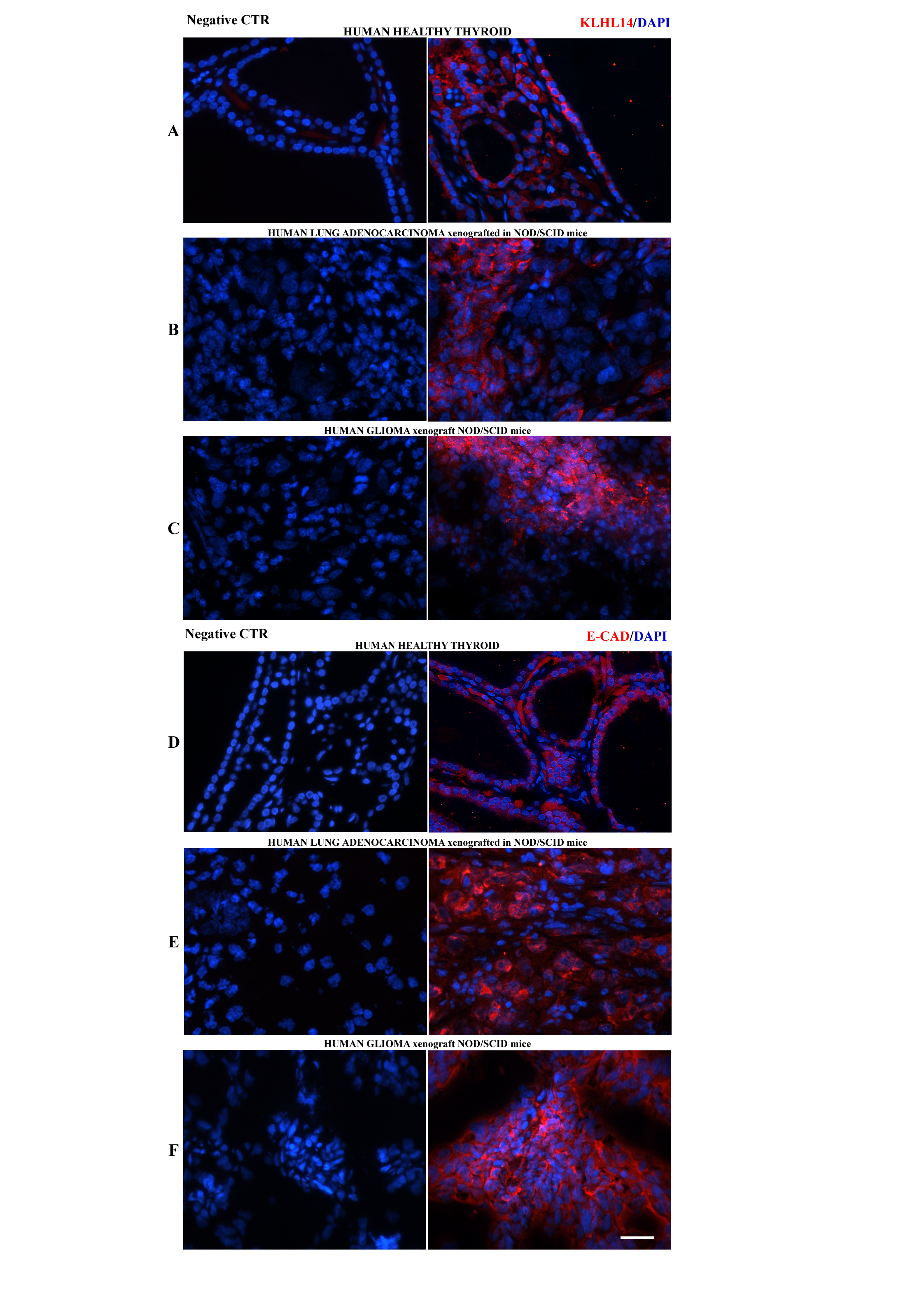

Supplement: Supplementary file 1 [file jcm-13-04409-s001.zip › Supplementary Figure S1.tif]

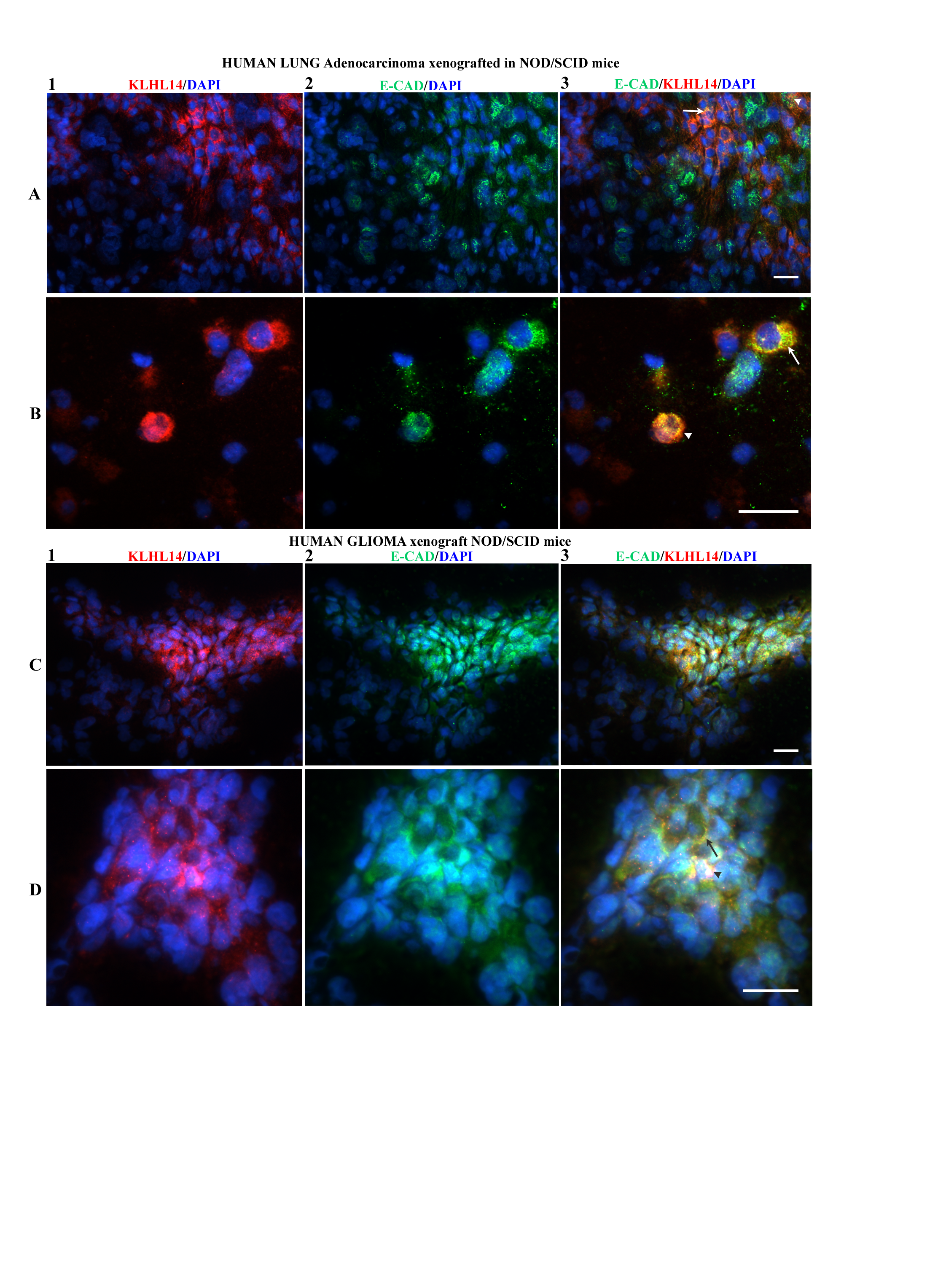

Supplement: Supplementary file 1 [file jcm-13-04409-s001.zip › Supplementary Figure S2.tif]

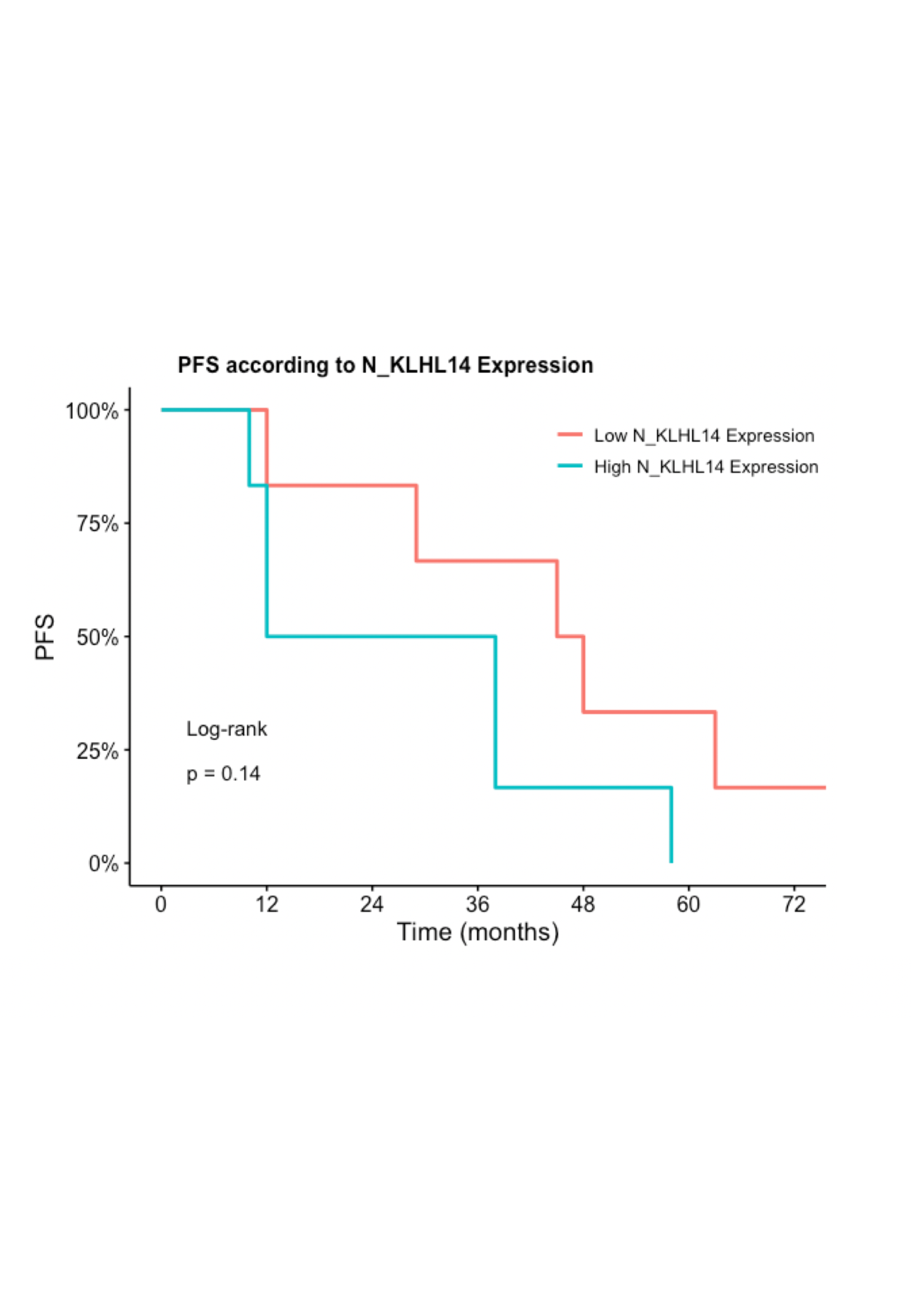

Supplement: Supplementary file 1 [file jcm-13-04409-s001.zip › Supplementary Figure S3.tiff]
